# Doxycycline release and antibacterial activity from PMMA/PEO electrospun fiber mats

**DOI:** 10.1590/1678-7757-2018-0663

**Published:** 2019-10-07

**Authors:** Luana Dutra de Carvalho, Bernardo Urbanetto Peres, Hazuki Maezono, Ya Shen, Markus Haapasalo, John Jackson, Ricardo M Carvalho, Adriana P Manso

**Affiliations:** 1 The University of British Columbia, Faculty of Dentistry, Department of Oral Biological and Medical Sciences, Vancouver, Canada; 2 The University of British Columbia, Faculty of Dentistry, Department of Oral Health Sciences, Vancouver, Canada; 3 Osaka University Graduate School of Dentistry Department of Restorative Dentistry and Endodontology, Osaka, Japan; 4 The University of British Columbia, Faculty of Pharmaceutical Sciences, Vancouver, Canada

**Keywords:** Antibacterial, Doxycycline, Drug-release, Electrospinning

## Abstract

**Objective:**

To investigate the use of polymethyl methacrylate (PMMA) electrospun fiber mats containing different amounts of polyethylene oxide (PEO) as a doxycycline delivery system and to test antibacterial activity against an oral pathogen.

**Methodology:**

PMMA powders or PEO (mol wt 200 Kd) (10,20,30% w/w/) were dissolved in N, N-dimethylformamide (DMF) to obtain a final polymer concentration of 15% in DMF (w/v). 2% Doxycycline monohydrate was added to the solutions and submitted to vortex mixing. The solution was transferred to a plastic syringe and fit into a nanofiber electrospinning unit. The parameters applied were: voltage at 17.2 kV; distance of 20 cm between the needle tip and the collector plate; target speed at 2 m/min; and transverse speed at 1cm/min. Syringe pump speed was 0.15 mm/min. The drug release analysis was performed by removing aliquots of the drug-containing solution (in PBS) at specific periods. Doxycycline release was quantified using RP-HPLC. Fiber mats from all groups had their antibacterial action tested against *S. mutans* based on inhibition halos formed around the specimens. The experiments were performed in triplicate. Gravimetric analysis at specific periods was performed to determine any polymer loss. Morphological characterization of the electrospun fibers was completed under an optical microscope followed by SEM analysis.

**Results:**

The addition of PEO to the PMMA fibers did not affect the appearance and diameter of fibers. However, increasing the %PEO caused higher doxycycline release in the first 24 h. Fibers containing 30% PEO showed statistically significant higher release when compared with the other groups. Doxycycline released from the fibers containing 20% or 30% of PEO showed effective against *S. mutans*.

**Conclusion:**

The incorporation of PEO at 20% and 30% into PMMA fiber mat resulted in effective drug release systems, with detected antibacterial activity against *S. mutans*.

## Introduction

Electrospinning is an electrostatic fiber fabrication technique. It has been subject of studies due to its versatility and potential for applications in diverse fields, including tissue engineering, biosensors, filtration, wound dressings, drug delivery, and enzyme immobilization.^1^,^2^ The sub-micron range spun fibers produced offer various advantages, such as high surface area to volume ratio, tunable porosity (when spun into a mat) and ability to manipulate nanofiber composition to get the properties desired.^1^

Recently, nanofibers have been used in different Dentistry fields, including composite reinforcement,^3^,^4^ periodontal regeneration,^5^ and implant surface treatments.^6^ They are also used as scaffolds for dental tissue and bone regeneration.^7^ However, the greatest potential of these fibers in Dentistry is the use of mats as drug delivery systems. The ability to produce drug-loaded polymeric fiber meshes or mats may offer an enhanced drug release profile compared with other polymeric drug release implants. As in many other fields, this mechanism can be convenient in Dentistry in multiple contexts.^8^

Among several polymers able to form ultrafine fibers in the electrospinning process, polymethyl methacrylate (PMMA) is a reliable biocompatible option, commonly used in restorative Dentistry.^9^ Electrospinning a blend of PMMA and polyethylene oxide (PEO) may result in biocompatible and toughness-enhanced ultra-thin fiber mats very useful for biomedical applications.^10^ PEO is frequently used as a co-spinning component in electrospinning solutions to offset problems related to continuous fiber formation without structural imperfections^11^ or even as a plasticizer to allow fiber formation when blended with different polymers, such as cellulose acetate.^12^

Doxycycline is an antimicrobial drug proved effective against oral bacteria, especially for periodontal disease.^13^ It presents anti-collagenase activity^14^ as additional advantage, which could be beneficial when treating sites sometimes compromised by undue metalloproteinase (MMP) activity such as at bonded interfaces with dentin.^15^

The described polymers formed into fibers loaded with antibacterial and anti-enzymatic drugs, as doxycycline, are expected to be an important controlled drug delivery tool in Dentistry. One considered that the addition of PEO would not affect the homogeneity or diameter or PMMA fibers formed into mats, but that the inclusion of a water soluble polymer such as PEO might increase drug release rates and antibacterial activity of the mats. Thus, the main objectives of this study were to produce PMMA fiber mats using two distinct PMMA powders; and to investigate whether the addition of PEO (200000 Dalton molecular weight) in different concentrations might enhance the release of an antibacterial and anti-MMP drug, the doxycycline.

## Methodology

### Fiber mat production

Polymer fiber mats were produced with two polymethyl methacrylate powders with different molecular weights (PMMA, Mw ∼75,000 and Mw∼996,000, Sigma-Aldrich, St. Louis, MO, USA), dissolved in N, N-dimethylformamide (DMF, Fisher Scientific, Waltham, MA, USA). Polyethylene oxide (PEO, Mw ∼200,000, Sigma-Aldrich, St. Louis, MO, USA) was added to the PMMA-DMF solution according to the concentration determined for each group (0%, 10%, 20%, or 30% of PEO). The final polymer concentration was 15% (w/v) (Table 1). Each PMMA-PEO-DMF mixture was stirred for 1 h, at 60°C to guarantee the dissolution of the polymers. The solution was maintained on the bench at room temperature. Doxycycline monohydrate (Sigma-Aldrich, St. Louis, MO, USA) was added to a final concentration of 2% w/w and submitted to vortex mixing for 1 min at 3000 rpm. Subsequentially, the vials were left for 10 minutes on the bench top at room temperature before electrospinning. The solution was transferred to a 10 mL plastic syringe and fit into a nanofiber electrospinning unit (NEU – Kato Tech, Japan). Electrospinning manufacture of nanofibers depends on many parameters, including the polymer molecular weight and concentration, flow rate, needle/collector distance, target speed, and applied voltage. Every polymer-drug combination needs to be spun under different conditions to achieve an optimal fiber production. This is usually optimized visually whereby the operator begins with intermediate values for these factors and sees how the polymer spins. In this study, a 15% polymer solution was associated with non-sputtering spinning and uniform fiber production, which could be seen to “draw” off the needle bead and a white mat appeared on the collection drum. Dry mat samples were collected and observed under high magnification optical microscopy (Olympus Model CX41RF) for uniformity using various flow rates and instrument settings to obtain an optimal fiber mat. The drug was then included in the polymer solution using optimal spinning settings. The final instrument settings were as follows: applied voltage at 17.2 kV; distance of 20 cm between the needle tip and the collector plate; target speed at 2 m/min; and transverse speed at 1 cm/min. Syringe pump speed was 0.15 mm/min to maintain a clear and visible Taylor cone. Temperature and humidity in the chamber were monitored. Air humidity was kept between 30% and 50% with the temperature held constant between 24 and 27 degrees Celsius. The collector plate was covered with an aluminum foil sheet. The fibers were produced over a period of 8 hours and collected randomly, without being aligned. The fiber mats formed were gently removed from the collector plate and stored in sealed plastic bags away from heat and humidity until their use.

**Table 1:** Concentrations of polymers, solvent, and drug added to the spinning solutions

Composition					
Groups	PMMA75k	PMMA996k	PEO200k	DMF	DOXY
0%PEO	0.875 g	0.875g	–	10 g	0.03g
10% PEO	0.79 g	0.79g	0.17 g	10 g	0.03g
20% PEO	0.70 g	0.70g	0.35 g	10 g	0.03g
30% PEO	0.615 g	0.615 g	0.52g	10 g	0.03g

### Doxycycline release profile

To analyze the doxycycline release rate from the fiber mats, 10 mg samples of PMMA-PEO-DOXY mats were immersed in vials with 2 mL of phosphate-buffered saline pH 7.4 (10 mM PBS), which were capped and placed in an incubator at 37°C with slow orbital shaking in the dark. The drug release was analyzed by removing 0.7 mL aliquots of drug-containing solution of each vial, in specific periods (1 h, 4 h, 8 h, 24 h, 48 h, 168 h). After each sampling, remaining media was replaced with 2 mL of fresh PBS. Doxycycline release was quantified using reverse phase High-Performance Liquid Chromatography (with a Waters chromatography system with Millennium software control). The chromatography system used a Novapak C^18^ column, a 20-μL injection volume, detection at 260 nm, and a mobile phase composed of 90% pH 3.4 potassium phosphate buffer and 10% acetonitrile, at a flow rate of 1 mL/min. Calibration graphs were linear in the 6–100 μg/mL concentration range.

### Gravimetric analysis

To measure the relative amount of PEO polymer lost from the mats in aqueous conditions, triplicate samples of each mat were cut in a square format (1×1 cm) and weighed on an analytical balance (Sartorius ME235S Genius). Thereafter, each sample was submerged in 10 mL of distilled water, maintained in an incubator at 37°C for one of the following periods: 1 h, 4 h, 8 h, 24 h, 48 h, and 168 h. After each period, the samples were removed from water and dried in an incubator at 60°C for 24 h. At that point, the remaining samples had their media discarded and 10 mL of distilled water was added to each vial. The dried samples were weighed, and the numbers were compared with the initial weight. Polymer loss was estimated based on the percentage of the film weight loss for each group.

### Antibacterial assay

*Streptococcus mutans* (NTCC# 10449) was used in this study. *S. mutans* bacterial suspensions was prepared with removal of 20 mL of *S. mutans* from the stock (frozen at −80°C). Bacteria were cultured overnight in 5 mL of brain heart infusion broth (BD ^™^ Bacto ^™^ BHI, Fischer Scientific, Waltham, MA, USA), at 37°C under aerobic conditions. After that, 20-50 µL of the overnight culture was put into 8 mL of BHI broth to obtain a second overnight growth solution. For the antibacterial test, 500 µL of bacterial solution (diluted 100×) was plated on the new BHI agar plate and let dry at room temperature for 1 h. Fiber mat discs with 6 mm diameter were positioned over the smeared bacteria and incubated overnight at 37°C. Filter paper (6 mm diameter) soaked in water and PMMA mats with no drugs were used as negative controls. Filter paper with doxycycline solution (concentration 300 µg/mL) was used as positive control by soaking the filter paper into the solution and directly applying it to the agar plate with the bacteria. Fiber mats with different concentrations of PEO and the same amount of doxycycline had their antibacterial action tested based on inhibition halos formed around the specimens. After the incubation period, the inhibition zones were measured. The experiments were performed in triplicate.

### Morphological characterization

Fiber characteristics were observed under an optical microscope (Olympus Model CX41RF). Scanning electron microscopy (SEM S-238ON, Hitachi, Tokyo, Japan) was used to evaluate the final electrospun mats at an acceleration voltage of 10 kV (SEM S-238ON, Hitachi, Tokyo, Japan). The control mats and the mats used in the release experiment were analyzed to evaluate alterations in fiber structures during drug/polymer release process. Randomly selected areas of each mat were cut in 5x5 mm squares and mounted on a stub with carbon tape (n=3). The stubs were then coated with platinum/palladium (Pt/Pd) with an ion sputter coater (Hitachi E-1030 Ion Sputter Coater, Hitachi, Tokyo, Japan). Random images were taken from the selected pieces of each mat at a magnification of 5000×. Average fiber diameter was estimated based on 15 random measurements from pictures taken at the same magnification with image software (Image J, NIH, USA).

### Statistical analysis

Statistical analysis of the data for drug release, weight loss, drug remaining, inhibition halo diameters and fiber diameter were investigated by one-way ANOVA (for each experiment). Level of significance of α=5% was set for all statistical analysis. Sigma Plot software (Systat Software, San Jose, USA) was also used.

## Results

### HPLC analysis

**Figure 1:**
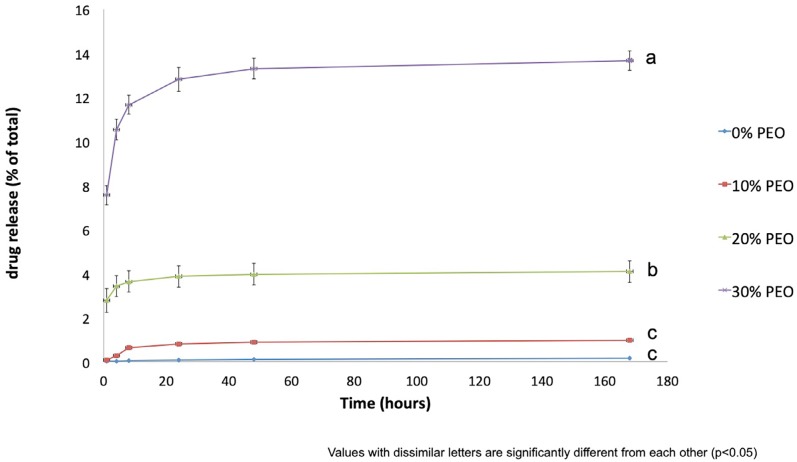
Release of doxycycline from PMMA fibers +/- PEO at 0%, 10%, 20%, or 30% loading

Insignificant drug release from PMMA (No PEO content) films was observed. The release of doxycycline from the different films was characterized by a burst phase over the first 24 h, followed by a slow release over the rest of the release study (Figure 1). The inclusion of PEO allowed concentration-dependent increase in doxycycline release from PMMA films. By 24 hours, the films had released approximately 1 ug, 8 ug, or 26 ug of loaded drug (for 10%, 20%, and 30% PEO content films, respectively). After 48 hours, the amount of doxycycline released was very low for all films, but the 30% PEO loaded films still released approximately 1 ug of drug, more than the other two films. The differences in doxycycline release between the fibers containing 10% PEO and 20% PEO compared with 30% PEO loaded films were statistically significant at all periods up to 48 hours (also, 20% PEO was higher than 10% PEO).

### Gravimetric analysis

**Figure 2:**
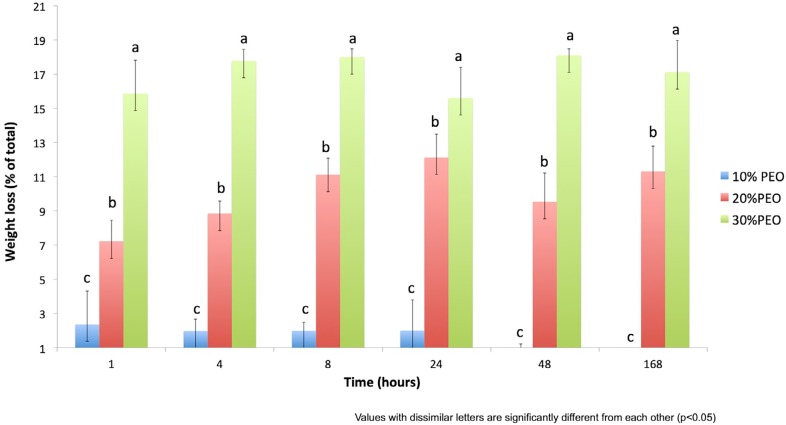
Percentage of weight lost from the PMMA fibers +/- PEO at 10%, 20%, or 30% loading

All fiber mats lost weight over time when placed in water (Figure 2). For 10% PEO loaded films, weight loss occurred in less than one hour with no further weight loss after that. For 20% PEO loaded mats, weight loss increased over 8 hours and then plateaued with no further loss. Similarly, for 30% loading, weight loss was complete at 8 hours. As PMMA is water-insoluble, the assumption was that all weight loss occurred due to PEO release. For 10% PEO loaded fiber mats, less than 20% of encapsulated PEO was released from the fibers. For 20% and 30% loadings, almost 50% of the PEO was released after 8 hours.

### Antibacterial analysis

**Figure 3:**
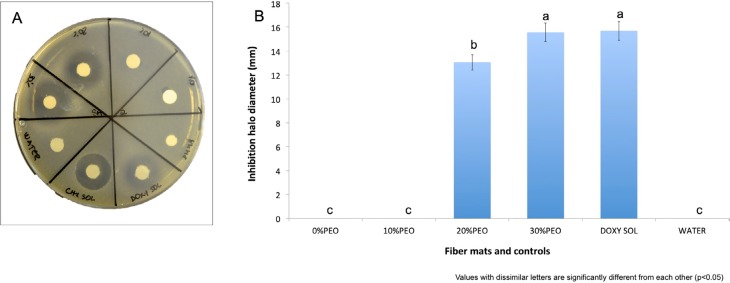
Comparison of inhibition halo size (mm) against *S. mutans* formed around the different fiber mats and controls. 3A: Image of inhibition halos; 3B: graphic representation of inhibition halo measurements (mm)

The initial concentration estimated for *S. mutans* was 5.0×10^8^ CFU/mL. Both 20% and 30% PEO loaded doxycycline fibers inhibited the growth of *S. mutans* as seen in Figure 3. These inhibition zones were similar to those resulting from non-encapsulated doxycycline in solution from filter paper.

### Morphological characterization

**Figure 4:**
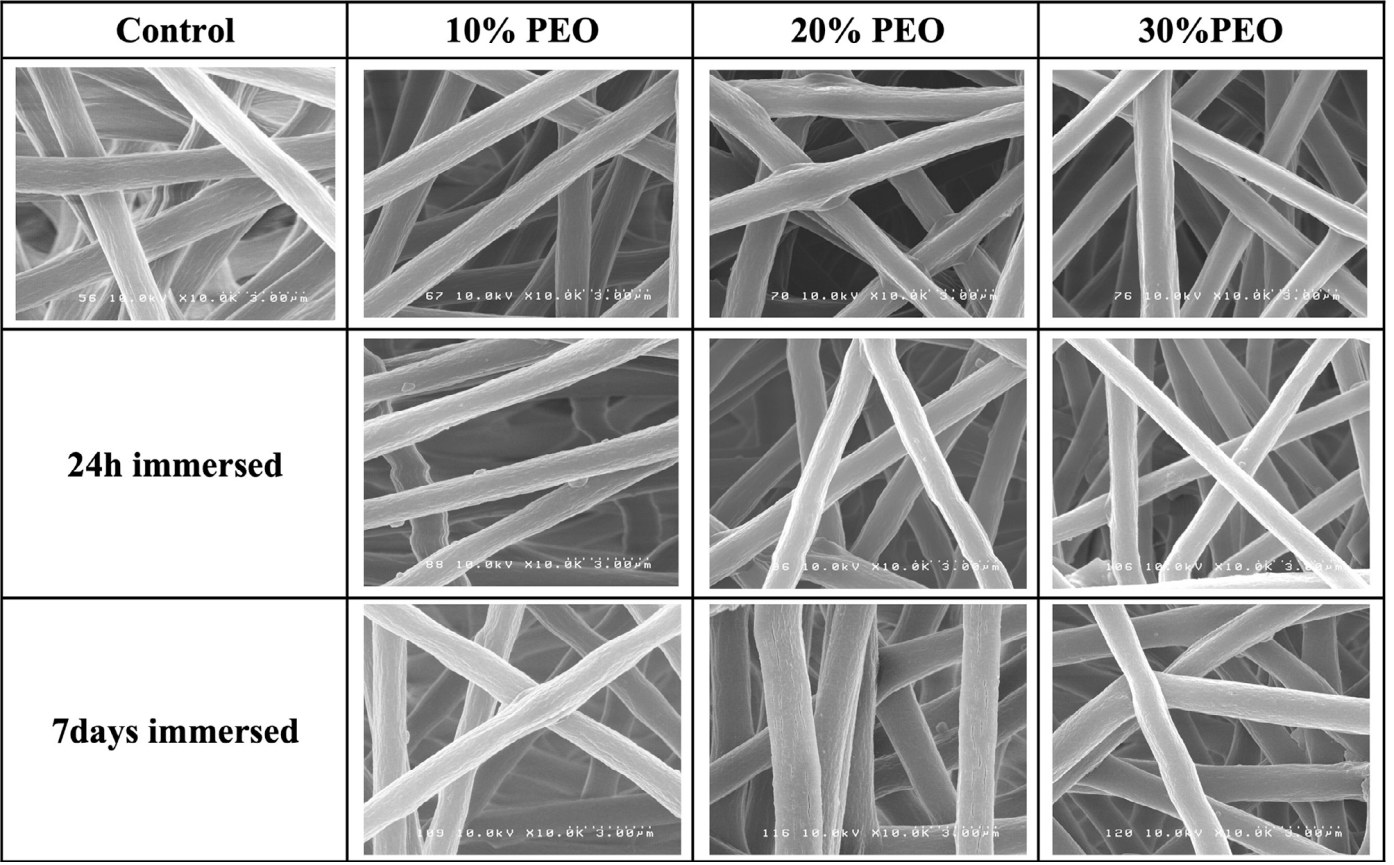
Scanning electron micrographs of PMMA control fibers (top left) and PMMA fibers containing 10%, 20%, and 30% PEO and 2% doxycycline before immersion in PBS, and after 24 hours and 7 days immersed (in sequence from top to bottom)

**Figure 5:**
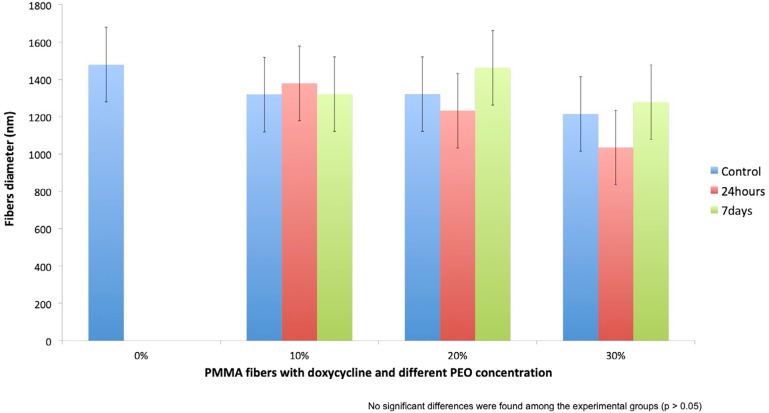
Fiber diameter (nm) with different PEO concentrations at post-spinning, 24 hours, and 7 days water immersion

SEM images showed randomly oriented fibers in all groups (Figure 4). All PMMA fiber mats presented homogenous fiber distribution with a uniform diameter and no drug aggregates or imperfections. The mean fiber diameter (standard deviation) of all groups is presented in Figure 5. One-way ANOVA showed the different PMMA/PEO ratio and immersion in PBS for up to 7 days did not affect significantly fiber diameter. (Figure 4 and Figure 5)

## Discussion

This study showed the doxycycline release profile was significantly affected by PEO amount present in PMMA fibers.

The manufacture and characterization of doxycycline loaded PMMA fiber mats by electrospinning are described in this study. Although other methods, such as pressurized gyratory spinning, are described as efficient methods for drug delivery purposes^16^, the method chosen provides an efficient encapsulation for the polymer PMMA, which is extensively used for dental applications. The electrospinning used in our laboratory allowed control of manufacturing variables for fiber optimization and mat production. Therefore, this method was selected for the manufacture of doxycycline fiber mats.

Generally, the incorporation of high molecular weight PEO polymer does not interfere with fiber production, since the long molecular weight molecules may align and help to electrospin the long PMMA molecules. At PMMA to PEO ratios of 70:30, the fiber chains (and mats) were strong and uniform in nature, demonstrating that high PEO incorporation levels might be achieved.^17^ FTIR is frequently used to characterize polymer blends but, in this case, the strong IR band arising from the C-O group is present in both PMMA and PEO. Reddy, et al.^18^ (2017) showed that the incorporation of PEO in PMMA films only resulted in a minor broadening of C-O stretch band at 1152 cm^−1^ wavenumber and that other variations in IR spectra were minor. Therefore, FTIR analysis was not performed in this study. In general, water-soluble antibiotics release very slowly from PMMA. PEO is reported as a carrier polymer that provides molecular entanglement required for electrospinning. Jackson, et al. showed the addition of water soluble molecules, such as sodium chloride, dextran or PEO, to PMMA bone cement monolithic blocks only caused minor changes in strength, but greatly enhanced the drug release profiles, such as vancomycin and linezolid.^19^ In another study, the addition of PEO to drug loaded polycaprolactone implants enhanced drug release profiles as the PEO dissolved out of hydrophobic brittle polycaprolactone.^20^ Whilst the insertion of PEO into cement is easy (simple mixing), electrospinning methods might be excessively compromised by the addition of large amounts of excipients. Electrospinning PMMA with PEO for enhanced drug release purposes overcomes a technical hurdle with positive results.

To the best our knowledge, no reports showed the addition of different percentages of PEO might affect doxycycline release profile from electrospun fibers. The hypothesis is that the release of significant PEO amounts from PMMA might open up the matrix slightly, so that water can access deeper fiber areas to solubilize and release doxycycline. Blended fibers using hydrophobic and hydrophilic polymers as PMMA and PEO may produce a prolonged and controlled release system for other drugs.^21^,^22^ The large surface area, associated with spun fabrics, allows fast and efficient solvent evaporation, providing incorporated drug limited time to recrystallize, which favors the formation of amorphous dispersions or solid solutions of drug in polymer.^23^

PMMA is a well-known polymer, commonly called acrylic, used for temporary crowns, complete or partial dentures, orthodontic appliances, and personalized trays for impressions among its dental applications. Its qualities of biocompatibility, reliability, relative ease of manipulation, and low toxicity were soon seized upon and incorporated by many different medical specialties.^24^,^25^ PMMA is hydrophobic and dimensionally stable. Therefore, it is a versatile polymer in drug delivery systems and has been used in various areas of the biomedical field: it has been proven to be an efficient antimicrobial agent delivery device, preventing *in vivo Staphylococcus aureus* biofilm formation.^26^ On the other hand, as this is a hydrophobic polymer, it does not swell at all in water. Other biocompatible polymers, such as poly (lactic-co-glycolic) (PLGA), are more commonly used in electrospinning methods for drug delivery because they swell in water and release drugs over a long period, until degradation.^27^,^28^. However, polymers that distort in water are not suitable for most dental applications requiring mechanical stability.

In this study, we showed little change in fiber diameter after the immersion of mats in water, making even 30% PEO loaded mats suitable for producing dimensionally stable fibers.

PEO is a polymer with high solubility in both aqueous and organic solvents. It is suitable for biological applications mostly because of its water solubility and low intrinsic toxicity.^29^ The high hydrophilic nature of PEO may increase the permeation of drugs when conjugated with them. It also enhances the physical and chemical stability of drugs and prevents the aggregation of drugs *in vivo* because of steric hindrance and/or masking of charges provided through the formation of a conformational cloud.^30^ Due to those characteristics, PEO was selected and suggested to act as a drug delivery polymer in this study.

The association of PMMA with other polymers, such as PEO, polyvinyl alcohol, and polycaprolactone (PCL), to achieve an ideal drug release profile has been previously shown.^10^,^31^,^32^ This study tested different PMMA/PEO ratios and their impact on doxycycline release profile. It was observed that doxycycline release increased as the percentage of PEO in fibers increased. Doxycycline release increased significantly when 30% PEO was added. When 10% PEO was added, the release was not significantly different from that of the control (0% PEO). It suggests doxycycline remained trapped within the fibers and strongly indicates the requirement of a minimal amount of PEO in fiber to allow the release of significant amounts of doxycycline. The relative contribution and the crucial role of PEO dissolution in the release of doxycycline correlates with the lower weight loss observed for the 10% PEO group.

Morphological characteristics of the fibers, such as diameter, may have a significant impact on the drug release.^33^ In this study, it was initially challenging to produce uniform, beadless fiber mats from PMMA. A pilot study revealed that the best formulation combined two different molecular weights of PMMA (75K and 996K) in pure DMF. The electrospun fibers formed were uniform, well distributed, and beadless. The fiber mats obtained were easy to handle. No statistically significant difference was found in the mean diameter among the groups. Although the fibers were relatively large in diameter (ca.>1000 nm) considering that nano level fibers can be produced, they were uniform among the groups and allowed us to interpret our findings based exclusively on the PEO concentration variable. Reproducibility and uniformity are more important than reduced diameter.^9^

Multiple alternative strategies to the use of antibiotics have been applied to enhance antimicrobial activity of polymeric fiber meshes, including the incorporation of antimicrobial metals, semi-metals, or graphene compounds.^34^,^35^. In this study, doxycycline was the drug selected for the experiment because it is a broad-spectrum antibiotic drug, which has been shown to be active against both gram-positive and gram-negative organisms. *Streptococcus mutans* was selected due to its clinical relevance on metabolic-induced dental tissue loss. Doxycycline has been successfully used to control the progression of periodontal disease by systemic and localized delivery at antimicrobial levels. The drug has also been encapsulated within nanotube-modified dentin adhesive, presenting sub-antimicrobial concentrations and ability to inhibit MMPs.^13^,^36^–^39^

In future studies using PMMA/PEO fiber, drug release profiles for specific dental applications need to be better quantified. However, the concept tested herein can open multiple doors for future studies aiming at validating the clinical application of doxycycline loaded PMMA-PEO mats as drug delivery systems. An example of a good clinical application for these mats could be their use as pulp protection and antibacterial materials for restorative procedures on deep cavities, protecting the pulp tissue and, simultaneously, acting as antibacterial and anti-MMP agent. Currently, an option for this purpose relies on calcium hydroxide, which may not provide an effective long-term seal against bacterial factors.^40^ This method would be an alternative approach as the material could release not just antibiotics, but also inductors of calcification or anti-inflammatory agents in future productions.

## Conclusion

Based on the experiments performed, one can conclude that consistent production of PMMA/PEO eletrospun fiber mats with adequate handling properties to be used in dental applications was possible. PEO inclusion influenced positively the doxycycline release profile from PMMA fiber mats, but did not alter the morphological characteristics of the fibers. PMMA fiber mats containing 20% and 30% PEO and 2% doxycycline presented antibacterial activity against *S. mutans*.
